# A specialized learner for inferring structured cis-regulatory modules

**DOI:** 10.1186/1471-2105-7-528

**Published:** 2006-12-05

**Authors:** Keith Noto, Mark Craven

**Affiliations:** 1 Department of Computer Sciences, University of Wisconsin, Madison, WI 53706, USA; 2 Department of Biostatistics and Medical Informatics, University of Wisconsin, Madison, WI 53706, USA

## Abstract

**Background:**

The process of transcription is controlled by systems of transcription factors, which bind to specific patterns of binding sites in the transcriptional control regions of genes, called *cis-regulatory modules *(CRMs). We present an expressive and easily comprehensible CRM representation which is capable of capturing several aspects of a CRM's structure and distinguishing between DNA sequences which do or do not contain it. We also present a learning algorithm tailored for this domain, and a novel method to avoid overfitting by controlling the expressivity of the model.

**Results:**

We are able to find statistically significant CRMs more often then a current state-of-the-art approach on the same data sets. We also show experimentally that each aspect of our expressive CRM model space makes a positive contribution to the learned models on yeast and fly data.

**Conclusion:**

Structural aspects are an important part of CRMs, both in terms of interpreting them biologically and learning them accurately. Source code for our algorithm is available at:

## Background

Eukaryotic transcription is controlled by multiple factors, which may need to bind to DNA in a specific arrangement in a gene's transcriptional control region. This type of regulation system is called a *cis-regulatory module *(CRM). The DNA motifs (specific patterns of nucleotides) to which these factors bind are often unknown, and may appear anywhere in a large region in the neighborhood of a gene. This region typically extends several thousand base pairs upstream of the transcription start site, and may also include DNA between the transcription start site and the start codon, and within introns of the transcribed gene. It is often the case that a set of genes are transcribed or expressed together under certain conditions, but the mechanisms underlying this co-expression are unknown. We would like a method that can aid in verifying that these genes are indeed transcribed by a common mechanism, and, more importantly, to explain this fact by finding the CRM which promotes transcription. The task we consider here is to learn such a CRM model from data. Specifically, given a set of sequences which are thought to contain a common cis-regulatory module and a set of sequences which are assumed not to, we wish to produce a description in terms of DNA binding sites which is true of the former set but not of the latter.

**Figure 1:**
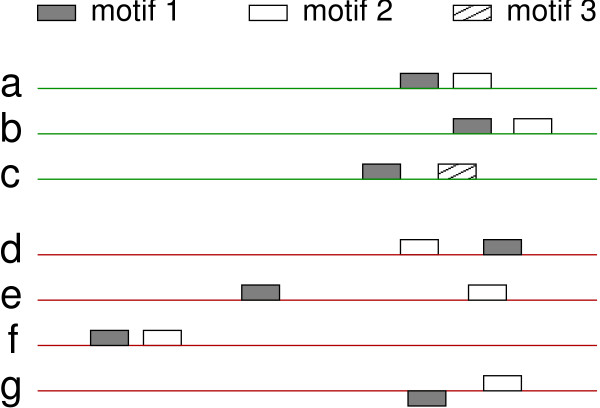
**Example CRM Learning Task**. An example of a CRM learning task: There are three types of transcription factor binding sites on these DNA sequences. Transcription will occur in a given condition if motif 1 is followed closely by either motif 2 or motif 3 near the transcription start site (right side) on the sense DNA strand. This is true for sequences **a**, **b**, **c**, but not sequences **d **(incorrect order), **e **(too far apart), **f **(too far upstream), and **g **(motif 1 on the incorrect DNA strand).

Several previous methods have characterized CRMs as a probabilistic over-representation of certain motifs within a window of predetermined size [1-4]. However, the models used are unable to represent very much about the physical arrangement among relevant binding sites. Given that a module consists of binding sites corresponding to multiple interacting transcription factors, we hypothesize that the relative locations of binding sites in real CRMs are an important consideration that the aforementioned models are unable to adequately represent. Consider the example in Figure 1. Suppose that a DNA sequence will be transcribed if, and only if, it contains transcription factor binding site motif 1 followed closely by either motif 2 or motif 3 near the start of transcription on the sense (template) strand. A CRM model that represents this situation must distinguish between DNA sequences that have it (a, b, and c) and those that do not because the motifs are out of order (sequence d), too far apart (sequence e), too far upstream of the start of transcription (sequence f), or bind to the wrong DNA strand (sequence g).

A few of these relationships can be represented by other previous methods. Sinha, *et al*. [3] point out that specific motifs may have strand preference, and their models do include a preference for a motif to *directly *follow another, but they do not describe arbitrary ordering constraints (*e.g*. a CRM includes all of motifs A, B, and C, and motif A must be most upstream), nor do they include strand preference into the CRM model itself. The models of Keleş *et al*. [5] are able to represent logical relationships (as in the example in Figure 1, motif 1 must be followed by motif 2 *or *motif 3), but they do not represent the ordering or proximity of the motifs. The models of Segal, *et al*., Aerts, *et al*., and Zhou, *et al*. model proximity between two motifs by whether or not they occur within a fixed-size window, but the motifs are otherwise independent and can occur anywhere in a transcription control region. The approach of Beer and Tavazoie [6] does capture motif orientation, and the relative order and distance between pairs of motifs. However, we argue that our models are more comprehensible than the probabilistic models of Beer and Tavazoie and that the increase in feature space that goes along with such a variety of relationships between possible binding sites requires that a learner take special steps to avoid overfitting.

We present a model representation which is able to describe logical relationships between binding sites, explicit upper-bounds on the distance between binding sites and between the CRM and the start of transcription (which may be known or estimated), the relative order (upstream or downstream) between any pair of binding sites, the DNA strand on which a binding site must appear, and a set of motifs which must *not *appear (*e.g*. the binding sites of *repressors*) in the CRM.

In order to make learning possible in such an expressive model space, we have developed a specialized learner which has two important distinctions: First, the search process is specifically tailored for the context of cis-regulatory modules. Second, although the expressivity capable of capturing all these physical aspects of a CRM is a major strength of our approach, only a few of these aspects may actually be needed to describe a given CRM. Therefore, there is a risk of overfitting due to this high-variance model space. For this reason, we have developed an expressivity selection method in which each aspect of the model space must be statistically justified by the data.

## Results and Discussion

### Representation

**Figure 2:**
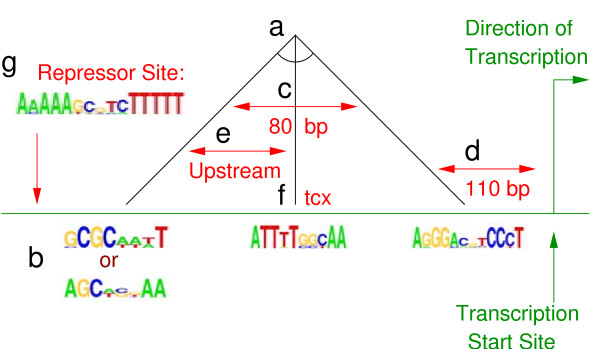
**Example CRM Model**. An example CRM model (motifs are represented here by sequence logos [14]). This particular model has three necessary binding sites (**a**), one of which can be satisfied by matching either of two motifs (**b**). One binding site must be within 80 bp of another (**c**), and the CRM must be within 110 bp of the start of transcription (**d**). One binding site must appear upstream of another (**e**), which must appear on the antisense DNA strand (**f**, denoted by tcx). Additionally, a certain motif must *not *appear upstream of the CRM (**g**).

Figure 2 illustrates an example of our CRM representation. We divide the expressivity of this representation into six distinct parts, which we will refer to as *structural aspects*:

1. *Multiple binding sites *(Fig. 2a). This is the basic structure of our representation.

2. *A multiplicity of motifs per binding site *(Fig. 2b). For each binding site, there is a set of motifs sufficient to represent it (*i.e*. connected with the logical or operator). We allow a binding site to be associated with multiple motifs because it may be the case that multiple transcription factors (with different binding motifs) may play the same role in a CRM, or a single transcription factor may have multiple or varying binding motifs.

3. *Distance constraints *(Fig. 2c,d). These specify a maximum distance (in base pairs) between the motifs that satisfy any two binding sites, or between the CRM and the transcription start site.

4. *Order constraints *(Fig. 2e). These specify that the motif that satisfies a particular binding site must be upstream of another binding site.

5. *Strand constraints *(Fig. 2f). These specify that a particular binding site must be on a specific DNA strand (In Fig. 2, tcx denotes the antisense–transcribed–strand).

6. *Repressor sites *(Fig. 2g). These specify that a particular motif (or any member of a disjunction of motifs) must not appear in the CRM, and its effective location can be constrained to be between a particular pair of binding sites, upstream of the CRM, or between the CRM and the transcription start site (in the case in Fig. 2, a single motif that must not appear upstream of the CRM).

### Learning a Model

Our learning algorithm learns a CRM model from positive and negative example sequences, a set of potential binding site motifs, and an evaluation function.

Positive examples are those believed to contain a shared CRM (*i.e*. a set of particular binding sites and structural aspects). These may be, for instance, the set of promoter sequences from a set of genes which are co-expressed under certain conditions and suspected to be co-regulated.

Negative examples are those believed not to contain the target CRM, although they certainly may contain other arrangements of motifs. The purpose of these sequences is to make the learned CRM model discriminative-so that it captures something specific to the given set of positive examples instead of something that is trivially or generally true of promoter sequences. The regulation of these negative examples may be related to the positive set in some interesting way (*e.g*. they are co-expressed under some other conditions), or they may simply be promoter sequences believed not to be regulated along with the positive examples.

The set of potential binding sites is specified by indicating the location of each occurrence in the positive and negative example sequences. These potential binding sites may come from a set of known or postulated transcription factor binding sites (*e.g*. from a database) or they may come from a standard motif-finding algorithm, such as MEME [7].

Pseudocode illustrating our learning algorithm is given in Table 1.

**Table 1:** Train Function

TRAIN(*trainset*, *aspects*, *phases*, *metric*, *K*)
1 *queue *← {**NULL_ SOLUTION**}
2 *CRM *← **NULL_SOLUTION**
3 **for ***phase *∈ *phases*
4 **while ***queue *is not empty
5 *current *← POP(*queue*)
6 **for **each applicable CRM *change *in *aspects *allowed in *phase*
7 *alt *← APPLY(*change*, *current*)
8 **if **there is a sufficiently low χ^2 ^test probability that the *trainset*
9 predictions of *current*, *alt *are from the same distribution
10 **then **insert *alt *into *queue*
11 sort *queue *by *metric*
12 limit *queue *to *K *solutions.
13 **if ***current *has a better score than *CRM *given *trainset*, *metric*
14 **then ***CRM *← *current*
15 repopulate *queue *with the best *K *solutions from *phase*
16 **return ***CRM*

The Train function takes: *trainset*, a set of labeled DNA sequences; *aspects*, a list of CRM aspects which can be included (*i.e*. the maximum number of binding sites, whether or not distance constraints are allowed, *etc*.); *phases*, a list of phases, specifying the set of model changes allowed in each; *metric*, a CRM model scoring metric; and *K*, a maximum queue size (beam width). For each phase, Train searches from the current list of solutions by making the changes allowed in that phase. It returns the best CRM model it finds.

Given sets of positive and negative DNA sequences, a set of potential binding site motifs, and an evaluation function, our algorithm searches through the space of possible models in an attempt to optimize the score given by the evaluation function. The ideal model would be satisfied by all the positive examples and none of the negative examples, so the evaluation function should be some measure of how well a given CRM model distinguishes between the positive and negative examples.

The search process is a best-first beam search [8] that starts with the null solution (an unconstrained model with zero binding sites) and searches in phases, modifying the best available solution and keeping only a queue of the best *K *models. Once the queue becomes empty, the best *K *solutions found are carried over to the initial queue for the next phase. In each phase, we apply a subset of the following operators:

• A new binding site is added.

• For a given binding site, a new motif is added.

• The distance from the CRM to the transcription start site is constrained (to the best distance smaller than the current distance, according to the data and the scoring metric).

• For a given pair of binding sites, the distance between them is constrained.

• For a given pair of binding sites, their relative order is constrained.

• For a given binding site, a strand constraint is imposed.

• A repressor motif is added between the CRM and the transcription start site

• A repressor motif is added upstream of the CRM

• A repressor motif is added between a pair of binding sites.

There are user-defined limits on the maximum number of binding sites, motifs that can represent a binding site, and repressor motifs in a set.

Many of these slight changes to a solution will not affect its score (*e.g*. if a motif that does not appear in any sequence were added to the list of motifs for a particular binding site, the model would match exactly the same sequences). For this reason, we insist on some statistical difference between the set of sequences predicted by any of these changes. We use a χ^2 ^test to decide whether we can reject the null hypothesis that two sets of sequence predictions by two different models come from the same distribution. It is not necessary to insist on near certainty when selecting the test's level of confidence; we mean only to avoid filling up our queue with multiple copies of essentially the same solution. If the test indicates that they come from different distributions, we add the new solution to the queue. Otherwise, we discard it.

In our experiments, we use two phases for the TRAIN procedure, making most of the above changes during the first phase, but adding repressor motifs in the second phase (because we argue these repressors can only be correctly added within the context of a CRM structure which has already been developed).

### Controlling the Expressivity of a Model

Since the model space is expressive enough to represent many aspects of a CRM, we must address the potential for overfitting. We first identify the CRM *model space *appropriate for the data, and then search through this space for the correct CRM. To do this, we hold aside a *tuning set *of training sequences and select our expressivity by comparing the results of training a model first including, then leaving out an entire *aspect *of our CRM model design. We keep the more expressive model space if and only if the results *with *the aspect in question show both an improvement and a *statistically significant difference*. That is, we use an aspect of CRM expressivity if and only if doing so is statistically justified by the data. This way, we select only the expressiveness required by a specific CRM, and we can then retrain the model by searching through the appropriate model space. Pseudocode illustrating this procedure is shown in Table 2. Note that Select-Train is the main procedure which calls the entire Train procedure as a subroutine.

**Table 2:** Select-Train Function

SELECT-TRAIN(*trainset*, *tuneset*, *aspects*, *phases*, *metric*, *K*)
1 *CRM *← TRAIN(*trainset*, *aspects*, *phases*, *metric*, *K*)
2 **repeat**
3 *unjustified_ aspects *← { }
4 **for ***aspect *∈ *aspects*
5 *alt_CRM *← TRAIN(*trainset*, *aspects *– *aspect*, *phases*, *metric*, *K*)
6 **if **there is not a sufficiently low χ^2 ^test probability that the *tuneset *predictions of *CRM*, *alt_CRM*
7 are from the same distribution **or ***CRM *scores better on *tuneset *than *alt_CRM*
8 **then ***unjustified_aspects *← *unjustified_aspects *∪ *aspect*
9 *aspects *← highest scoring set resulting from removing one of *unjustified_ aspects *based on *tuneset*
10 *CRM *← *alt_CRM *associated with these aspects
11 **until ***unjustified_aspects *is empty
12 *final_CRM *← TRAIN(*trainset *+ *tuneset*, *aspects*, *phases*, *metric*, *K*)
13 **return ***final_CRM*

The Select-Train algorithm takes: *trainset*, a set of labeled DNA sequences; *tuneset*, held-aside evaluation data; *aspects*, a list of CRM aspects to consider; as well as *phases*; *metric*, and *K*, which are arguments to the Train algorithm. It removes aspects from the original list which are statistically shown (using the *tuning *set) not to contribute. Finally, it returns a CRM trained with all the data, using the CRM aspects chosen.

Since the inclusion of one model aspect may depend on another (*e.g*. distance constraints are only effective once the affected binding sites are identified), we do backward selection instead of forward. That is, we start with the full set of CRM structural aspects, and then remove some as is appropriate as opposed to starting with an empty set and adding to it. The list of model space restrictions is:

• Reduce the maximum number of binding sites by one.

• Reduce the maximum number of motifs (disjuncts) per binding site by one.

• Disallow distance constraints.

• Disallow order constraints.

• Disallow strand constraints.

• Reduce the maximum number of motifs in a set of repressor motifs by one.

Unless leaving the aspect in the model space produces a statistically significant improvement as determined by a χ^2 ^test, we remove it. If more than one restriction in the list above is being considered, we make the restriction that gives us the best tuning set score. That is, we only make one model space restriction at a time. This process is repeated on the more restricted model space until no more restrictions should be considered (all structural aspects are statistically justified). This approach is similar to backward feature selection [9,10]. However, we are not deciding on whether or not to include specific features (*e.g*. what is the distance between motif *A *and motif *B *in each DNA sequence), but rather we are deciding on whether or not to include entire *aspects*.

### Experimental Results

We test our approach on several data sets, summarized in Table 3. Three of these data sets have been used in previous studies of computational CRM finding. the Gasch *et al*. data set, however, is novel. In each case, we obtain upstream/promoter sequences from the University of California Santa Cruz Genome Browser [11] and perform cross-validation to evaluate our algorithms. We obtain a set of candidate motifs from running MEME [7] on the positive examples (not including any test sequences held-aside for evaluation) and from running MEME on on upstream/promoter regions randomly sampled from the appropriate organism. For the fly data set, we also evaluate our approach when it is provided with a set of known motifs [3,12].

**Table 3:** Data Sets

Data Set	Organism	Description
Lee *et al*.	*S. cerevisiae*	25 sets of genes with strong evidence (*p*-value ≤ 0.01) from the genome-wide location analysis of Lee *et al*. [15] that a specific pair of regulators bind to their upstream regions. This is a recreation of the data sets used by Segal *et al*. [2]. For each data set, we use 100 yeast promoters chosen at random as negative examples.
Gasch *et al*.	*S. cerevisiae*	Three sets of genes associated with environmental stress response (ESR) in Yeast, described in [16]. We use promoter sequences from non-ESR yeast genes as negative examples.
Sinha *et al*.-Yeast	*S. cerevisiae*	A set of six yeast sequences where MCM1 and MATα2 are known to bind, described in Sinha *et al*. [3]. For negative examples, we used nine promoter sequences which contain binding sites for *either *MCM1 or MATα2, but not both.
Sinha *et al*.-Fly	*D. melanogaster*	A set of eight fly genes associated with the gap gene system, described in Sinha *et al*. [3]. We use 10 kb promoter sequences, and 100 promoter sequences selected randomly from the fly genome to use as negative examples.

Summary of the data sets on which we test our algorithms

These motifs are described by position weight matrices (PWMs). We compare the likelihood of each PWM generating a subsequence in our data sets to the likelihood of the sequence being generated by a 5th-order Markov chain which is trained on the promoter regions of an entire genome. We consider a motif to be present if the ratio exceeds a threshold.

For our algorithm's scoring metric, we wish to measure how well the model predicts all, and only, the sequences which contain the target CRM. *Precision *is the frequency with which positive predictions are true positives, not false positives: P=TPTP+FP
 MathType@MTEF@5@5@+=feaafiart1ev1aaatCvAUfKttLearuWrP9MDH5MBPbIqV92AaeXatLxBI9gBaebbnrfifHhDYfgasaacH8akY=wiFfYdH8Gipec8Eeeu0xXdbba9frFj0=OqFfea0dXdd9vqai=hGuQ8kuc9pgc9s8qqaq=dirpe0xb9q8qiLsFr0=vr0=vr0dc8meaabaqaciaacaGaaeqabaqabeGadaaakeaacqWGqbaucqGH9aqpdaWcaaqaaiabdsfaujabdcfaqbqaaiabdsfaujabdcfaqjabgUcaRiabdAeagjabdcfaqbaaaaa@36BF@. *Recall *is the frequency with which the correct sequences are predicted as positive and are not false negatives: R=TPTP+FN
 MathType@MTEF@5@5@+=feaafiart1ev1aaatCvAUfKttLearuWrP9MDH5MBPbIqV92AaeXatLxBI9gBaebbnrfifHhDYfgasaacH8akY=wiFfYdH8Gipec8Eeeu0xXdbba9frFj0=OqFfea0dXdd9vqai=hGuQ8kuc9pgc9s8qqaq=dirpe0xb9q8qiLsFr0=vr0=vr0dc8meaabaqaciaacaGaaeqabaqabeGadaaakeaacqWGsbGucqGH9aqpdaWcaaqaaiabdsfaujabdcfaqbqaaiabdsfaujabdcfaqjabgUcaRiabdAeagjabd6eaobaaaaa@36BF@. We use F1 as our scoring metric, which is the harmonic average of precision and recall: F1=2×P×RP+R
 MathType@MTEF@5@5@+=feaafiart1ev1aaatCvAUfKttLearuWrP9MDH5MBPbIqV92AaeXatLxBI9gBaebbnrfifHhDYfgasaacH8akY=wiFfYdH8Gipec8Eeeu0xXdbba9frFj0=OqFfea0dXdd9vqai=hGuQ8kuc9pgc9s8qqaq=dirpe0xb9q8qiLsFr0=vr0=vr0dc8meaabaqaciaacaGaaeqabaqabeGadaaakeaacqWGgbGrcqaIXaqmcqGH9aqpdaWcaaqaaiabikdaYiabgEna0kabdcfaqjabgEna0kabdkfasbqaaiabdcfaqjabgUcaRiabdkfasbaaaaa@3A75@.

We set the maximum number of binding sites to three, the maximum number of motifs per binding site to three and the maximum number of repressor motifs in a set to one. We evaluate our model by using cross-fold-validation: We hold aside some data, train on the remainder, and then evaluate our trained models' predictions on the held-aside data. We predict that the held-aside sequence is a positive example if and only if it contains our hypothesized CRM. This process is repeated with different examples held aside for evaluation, and the results from each fold are summed together.

For each data set, we calculate an F1 score, ℱ
 MathType@MTEF@5@5@+=feaafiart1ev1aaatCvAUfKttLearuWrP9MDH5MBPbIqV92AaeXatLxBI9gBamrtHrhAL1wy0L2yHvtyaeHbnfgDOvwBHrxAJfwnaebbnrfifHhDYfgasaacH8akY=wiFfYdH8Gipec8Eeeu0xXdbba9frFj0=OqFfea0dXdd9vqai=hGuQ8kuc9pgc9s8qqaq=dirpe0xb9q8qiLsFr0=vr0=vr0dc8meaabaqaciaacaGaaeqabaWaaeGaeaaakeaaimaacqWFXeIraaa@3787@ (the same statistic as our algorithm's scoring metric), and use Fisher's exact test [13] to calculate a *p*-value. If our positive predictions (true positives plus false positives) were made simply by randomly sampling without replacement from the data set, this *p*-value would be the probability of an F1 score of ℱ
 MathType@MTEF@5@5@+=feaafiart1ev1aaatCvAUfKttLearuWrP9MDH5MBPbIqV92AaeXatLxBI9gBamrtHrhAL1wy0L2yHvtyaeHbnfgDOvwBHrxAJfwnaebbnrfifHhDYfgasaacH8akY=wiFfYdH8Gipec8Eeeu0xXdbba9frFj0=OqFfea0dXdd9vqai=hGuQ8kuc9pgc9s8qqaq=dirpe0xb9q8qiLsFr0=vr0=vr0dc8meaabaqaciaacaGaaeqabaWaaeGaeaaakeaaimaacqWFXeIraaa@3787@ or higher. If this *p*-value is sufficiently low (less than 0.01, following Segal and Sharan [2]), we consider our CRM for this data set to be significant.

Our results are shown in Table 4. We find a significant CRM in 17 of the 25 Lee *et al*. data sets (Table 3). In their similar experiments, Segal *et al*. found significant CRMs in only 12 of the 25 data sets (Note that the *p*-value calculations of Segal *et al*. are not identical to ours; as their CRM model makes probabilistic predictions, they are able to calculate a *p*-value using a *classification margin *[2]). Many of the motifs included in our CRM models correspond to known binding site motifs for the proteins thought to bind to the promoter regions in these data sets. However, we do not focus on recovered motifs, because our approach does not define these (they are found by MEME), it only selects them from a set of candidates. We find significant CRMs in the three Gasch *et al*. data sets, which suggests that our method can be used to find novel CRMs corresponding to genes clustered by expression analysis.

**Table 4:** Results

	Data set	True Positives	False Positives	True Negatives	False Negatives	Precision	Recall	F1 Score	*p*-value
Lee *et al*.	GAT3, RGM1	5	22	78	10	0.185	0.333	0.238	0.253
	GAL4, YAP5	7	16	84	9	0.304	0.438	0.359	0.0169
	GAT3, PDR1	10	9	91	7	0.526	0.588	0.556	**1.138e-05**
	CIN5, NRG1	10	18	82	8	0.357	0.556	0.435	**1.53e-03**
	RGM1, YAP5	7	13	87	11	0.35	0.389	0.368	0.0137
	NDD1, SWI4	15	23	77	7	0.395	0.682	0.5	**8.51e-05**
	SKN7, SWI4	12	38	62	10	0.24	0.545	0.333	0.118
	PDR1, YAP5	13	36	64	10	0.265	0.565	0.361	0.0585
	FKH2, SWI4	11	20	80	13	0.355	0.458	0.4	0.0113
	PHD1, YAP6	14	18	82	10	0.438	0.583	0.5	**1.52-e04**
	FHL1, YAP5	15	19	81	10	0.441	0.6	0.508	**1.07-e04**
	FKH2, MCM1	20	33	67	5	0.377	0.8	0.513	**2.45-e05**
	MBP1, NDD1	13	24	76	12	0.351	0.52	0.419	**7.52-e03**
	ACE2, SWI5	17	29	71	9	0.37	0.654	0.472	**7.97-e04**
	FKH2, MBP1	19	31	69	8	0.38	0.704	0.494	**2.60-e04**
	MCM1, NDD1	24	36	64	4	0.4	0.857	0.545	**2.37-e06**
	RAP1, YAP5	15	7	93	14	0.682	0.517	0.588	**3.68-e07**
	NRG1, YAP6	15	22	78	15	0.405	0.5	0.448	**3.72-e03**
	GAT3, YAP5	25	11	89	14	0.694	0.641	0.667	**8.75-e10**
	CIN5, YAP6	26	41	59	14	0.388	0.65	0.486	**8.47-e03**
	MBP1, SWI4	27	33	67	13	0.45	0.675	0.54	**2.00-e04**
	SWI4, SWI6	20	45	55	23	0.308	0.465	0.37	0.506
	MBP1, SWI6	39	38	62	5	0.506	0.886	0.645	**6.34-e09**
	FKH2, NDD1	35	83	17	15	0.297	0.7	0.417	0.978
	FHL1, RAP1	89	36	64	25	0.712	0.781	0.745	**3.33-e10**

Gasch *et al*.	iESR	89	453	4497	181	0.164	0.33	0.219	**~0**
	rESR_PACcluster	173	260	4690	255	0.4	0.404	0.402	**~0**
	rESR_RPcluster	50	84	4866	71	0.373	0.413	0.392	**~0**

Sinha *et al*.	Yeast	6	2	7	0	0.75	1	0.857	**5.59-e03**

Sinha *et al*.	Fly	3	3	97	5	0.5	0.375	0.429	**4.92-e03**
Sinha *et al*.	Fly, known PWMs	7	10	90	1	0.412	0.875	0.560	**5.10e-06**

Results of running our algorithm on the data in Table 3. Significant CRMs (*p*-value < 0.01) are indicated with bold text.

We find a significant CRM in the Sinha *et al*. fly data set as well. For this data set, using motifs suggested by MEME, we find three true positives and three false positives. Although this result is statistically significant, we hypothesize that the reason we are unable to recover more of the positive examples is because the training set size is too small for MEME to find good candidate motifs. To test this, we use the PWMs from Rajewsky, *et al*. and Sinha, *et al*. [3,12] and locate positions where these motifs are most likely to occur. In this case, we recover seven of eight positive examples. Note that we do not compare our results to those of Sinha *et al*. because we use this data set to evaluate predictive accuracy on held-aside data, whereas they do not.

We wish to determine whether or not the inclusion of structural aspects increases the accuracy of our models. We do this by comparing the results of our approach to those obtained when we limit the set of *aspects *given to the *Train *function. We do this in two ways: First, we measure this accuracy by the F1 score of our predictions on held-aside data, and compare these scores to those obtained by a restricted version of our algorithm, for which the only *aspect *given to the *Train *function is multiple binding sites. This experiment is designed to compare against the model space of several previous methods in which a CRM model is characterized simply by the presence of a set of motifs anywhere in an input sequence. We refer to this as the "bag-of-motifs" approach. Second, we compare the F1 scores of our approach to those of running our algorithm with a single structural aspect left out of the set given to the *Train *function, for each aspect/dataset pair. This is designed to determine whether each structural aspect by itself makes a positive contribution to the learned models. We refer to these experiments as "lesion tests."

**Figure 3:**
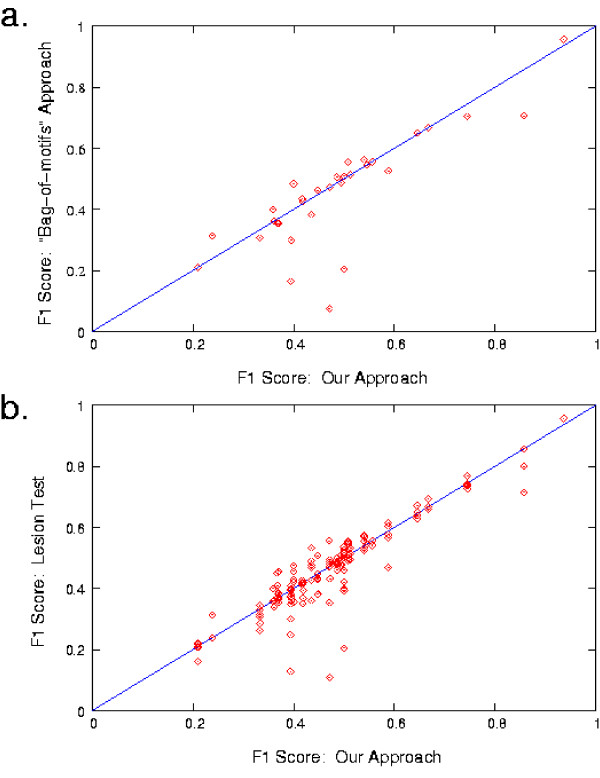
**Comparison to Models with Limited Expressivity**. **a**. The F1 score of our approach (x-axis) compared to the F1 score of the bag-of-motifs approach (y-axis). **b**. The F1 score of our approach (x-axis) compared to the F1 score of a lesion-test (y-axis) wherein a model was trained with one structural aspect left out of the set given to the *Train *function (this experiment is run for each aspect and for each data set).

**Figure 4:**
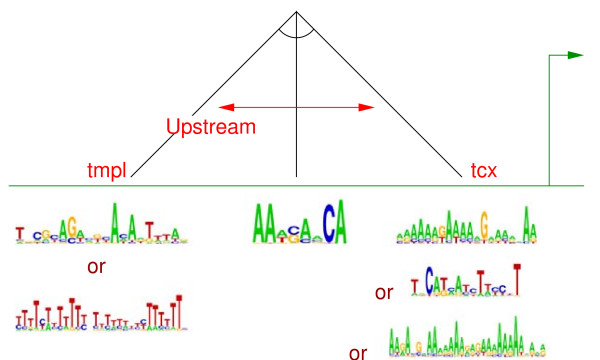
**Example CRM Hypothesis**. Hypothesized CRM for the PHD1, YAP6 (Lee *et al*.) data set. The relative order of two binding sites (characterized by a set of possible motifs) is constrained.

**Figure 5:**
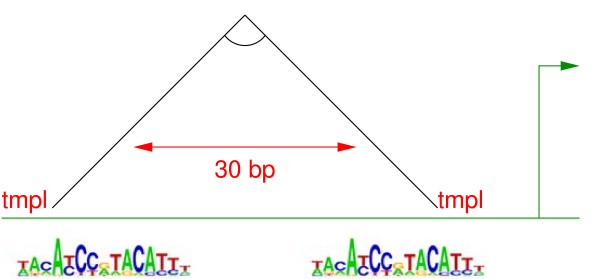
**Example CRM Hypothesis**. Hypothesized CRM for the rESR_RPcluster data set. The model consists of two copies of the same motif on the same strand and nearby one another.

These comparisons are illustrated in Fig. 3. Note that sometimes the inclusion of a structural aspect can lead to overfitting (a point slightly above the diagonal line), but often it is essential (a point well below the line). Indeed, considering all data sets, the F1 score is more often higher with all aspects included than it is when any single structural aspect is removed. On the 25 yeast data sets from Lee *et al*. (Table 3a), the bag-of-motifs approach is often about as accurate as our approach. One exception is shown in Fig. 4. Here, our algorithm discovers that the order of binding sites is important. Compare the test set F1 score of our approach (0.500) to that of the bag-of-motifs approach (0.205). On the other data sets, our approach scores much higher than the bag-of-motifs approach. For instance, Figure 5 shows the hypothesis CRM model for the data set, rESR_RPcluster, which involves distance and strand constraints. The bag-of-motifs hypothesis (not shown) also includes two copies of the same motif, but without structural constraints, the model accepts eight additional true positives, and 265 additional false positives. Using our approach on the Sinha *et al*.-Fly data set, we find three true positives and three false positives (compared to two true positives and 34 false positives using the bag-of-motifs approach). Using PWMs from the literature, we recover seven of eight positive examples, with 10 false negatives (compared to six true positives and 16 false negatives using the bag-of-motifs approach).

## Conclusion

One of the primary steps in gene regulation is transcription, and the ability to learn CRMs directly from data will be a crucial part of understanding how transcription is controlled. Our experiments, as well as those of Beer and Tavazoie [6] indicate that transcription is controlled not only by the presence of binding sites, but also by relationships between their locations. Our models represent a step forward in this area because these aspects are represented in a model which is easy to inspect and understand, and our results show that each of them contributes to the identification of significant CRMs in real biological data. With this increase in expressiveness, there is inevitably a risk of overfitting. We use data to identify the appropriate CRM aspects during the process of training our models. We believe that our novel approach of model space selection is an important and necessary step to facilitate the move toward more expressive models.

## Availability and Requirements

The source code for our algorithm is available at: .

## Authors' contributions

KN wrote the software and carried out the computational experiments. MC and KN designed the algorithm and experiments. All authors read and approved the final manuscript.
